# Charting infants’ motor development at home using a wearable system: validation and comparison to physical growth charts

**DOI:** 10.1016/j.ebiom.2023.104591

**Published:** 2023-05-01

**Authors:** Manu Airaksinen, Elisa Taylor, Anastasia Gallen, Elina Ilén, Antti Saari, Ulla Sankilampi, Okko Räsänen, Leena M. Haataja, Sampsa Vanhatalo

**Affiliations:** a BABA Center, Pediatric Research Center, Department of Clinical Neurophysiology, New Children's Hospital and HUS Imaging, Helsinki University Hospital, Helsinki, Finland; b Department of Materials Science and Engineering, Universitat Politècnica de Catalunya, BarcelonaTech, Terrassa, Spain; c Department of Paediatrics, Kuopio University Hospital and University of Eastern Finland, Kuopio, Finland; d Unit of Computing Sciences, Tampere University, Tampere, Finland; e Department of Pediatric Neurology, Children's Hospital, Helsinki University Hospital and University of Helsinki, Helsinki, Finland; f Department of Physiology, University of Helsinki, Helsinki, Finland

**Keywords:** Neurodevelopment, Motor development, Milestones, Out-of-hospital, Human activity recognition, Cerebral palsy

## Abstract

**Background:**

Early neurodevelopmental care and research are in urgent need of practical methods for quantitative assessment of early motor development. Here, performance of a wearable system in early motor assessment was validated and compared to developmental tracking of physical growth charts.

**Methods:**

Altogether 1358 h of spontaneous movement during 226 recording sessions in 116 infants (age 4–19 months) were analysed using a multisensor wearable system. A deep learning-based automatic pipeline quantified categories of infants' postures and movements at a time scale of seconds. Results from an archived cohort (dataset 1, N = 55 infants) recorded under partial supervision were compared to a validation cohort (dataset 2, N = 61) recorded at infants’ homes by the parents. Aggregated recording-level measures including developmental age prediction (DAP) were used for comparison between cohorts. The motor growth was also compared with respective DAP estimates based on physical growth data (length, weight, and head circumference) obtained from a large cohort (N = 17,838 infants; age 4–18 months).

**Findings:**

Age-specific distributions of posture and movement categories were highly similar between infant cohorts. The DAP scores correlated tightly with age, explaining 97–99% (94–99% CI 95) of the variance at the group average level, and 80–82% (72–88%) of the variance in the individual recordings. Both the average motor and the physical growth measures showed a very strong fit to their respective developmental models (R^2^ = 0.99). However, single measurements showed more modality-dependent variation that was lowest for motor (***σ*** = 1.4 [1.3–1.5 CI 95] months), length (***σ*** = 1.5 months), and combined physical (***σ*** = 1.5 months) measurements, and it was clearly higher for the weight (***σ*** = 1.9 months) and head circumference (***σ*** = 1.9 months) measurements. Longitudinal tracking showed clear individual trajectories, and its accuracy was comparable between motor and physical measures with longer measurement intervals.

**Interpretation:**

A quantified, transparent and explainable assessment of infants' motor performance is possible with a fully automated analysis pipeline, and the results replicate across independent cohorts from out-of-hospital recordings. A holistic assessment of motor development provides an accuracy that is comparable with the conventional physical growth measures. A quantitative measure of infants’ motor development may directly support individual diagnostics and care, as well as facilitate clinical research as an outcome measure in early intervention trials.

**Funding:**

This work was supported by the Finnish Academy (314602, 335788, 335872, 332017, 343498), Finnish Pediatric Foundation (Lastentautiensäätiö), Aivosäätiö, 10.13039/501100006306Sigrid Jusélius Foundation, and HUS Children’s Hospital/HUS diagnostic center research funds.

**Research in context**

**Evidence before this study**

An uncompromised infantile neurodevelopment is fundamental for a lifelong success. More than every tenth newborn infant has a medically identified neurodevelopmental risk that calls for an early neurodevelopmental follow-up and consideration of therapeutic interventions. An evidence-based development of neurodevelopmental care is critically dependent on measures of early motor development that should be reliable, objective and ecologically valid; however, there is scarcity of such methods in both clinical practice and in clinical research. It is well established that early gross motor development is characterized by acquiring new skills, which are commonly observed in an infant as reaching discrete motor milestones. Measuring infants' spontaneous motor activity over longer times in a native environment, such as the home, can provide a more extensive and detailed account of motor performance. Our recent development of infant wearables, comprising a multisensor garment with an automated, deep learning -based algorithmic assessment, has made it possible to quantify infant's motor performance at high accuracy. If successful, such measures could even support construction of “motor growth charts” to complement the world widely used physical growth charts based on weight, height or head circumference measurements. It is currently not known, however, whether measures from infant wearables are replicable across cohorts from different recording settings, or how do they compare in interpretative potential with the physical growth charts.

**Added value of this study**

We show a detailed validation of prior results from a controlled study setting by recruiting a prospective cohort of infants recorded at infant's homes by their parents. The novel cohort compares closely to a scenario of future clinical practice where infants' gross neuromotor performance is measured without supervision by the healthcare workers, and the recorded data is analysed using fully automated pipelines located in a cloud server. The findings show remarkable consistency in all measures between the cohorts. Moreover, we show that the accuracy of developmental measurements, estimated as prediction error of age, is highly comparable between measures of function (motor) and physical size (height, weight, head circumference; individually or combined). All of these measures support the idea that a longitudinal and quantitative tracking of infants' motor development is feasible at high accuracy.

**Implications of all the available evidence.**

A wearable solution of this kind can be readily implemented into health care or clinical research practice. Its automatically generated results offer intuitive, transparent, explainable, and quantified interpretation of motor development. The overall accuracy of such a chart of holistic gross motor development is comparable with the widely used physical growth charts. Individual metrics from the automated analysis outputs, such as measures of different posture and movement times, provide evolving potential for establishing reference charts in the future. These findings together support a vision that functional growth charts could be constructed as a complement to physical growth charts, implying significant improvement in tracking functional, or neuromotor development at an individual level. Such early outcome measures hold also promise for facilitating both clinical research and therapeutic trials.

## Introduction

Improvements in neurodevelopmental care are globally challenged by a scarcity of objective, reliable and scalable solutions for a quantitative assessment of early development.^1^ A particular challenge in the clinical research and practice is to track functional development of an individual infant in the presence of the large intra- and inter-individual variations. In the current practice, infant's motor abilities are assessed with an array of screening tools, such as questionnaires for motor milestones^2^^,^^3^ which are mostly qualitative and readily compromised by the natural variability in infant's motor development.^4^^,^^5^ The gold standard assessment is provided by standardized neurodevelopmental assessment batteries performed by trained professionals. They offer more fine-grained information^6^, ^7^, ^8^, ^9^ by collating sets of clinically observable or testable items. All of these methods are unavoidably subjective and at least partly qualitative; In addition, most tests are performed by professionals in unnatural situations from an infant's perspective, compromising their ecological validity. There is hence a demand to develop methods for early neurodevelopmental tracking that are robust to variability in infant physiology, the skills of the assessor, and the testing environment.^1^^,^^8^^,^^10^ One possible solution could be with an objective measurement of intrinsic movement behaviour at the home, the ecologically most valid environment.

Recent progress in sensor technology and signal analysis methods have made it possible to monitor extended periods of infants' movements during sessions of spontaneous free play, even in out-of-hospital settings.^11^, ^12^, ^13^, ^14^ Most previous studies have shown that quantitation of the total amount of movements is possible out-of-hospital,^12^ and classification of infants’ posture can be done reliably though practical challenges may remain in at-home recordings.^14^ However, more recent development of comfortable multisensory wearables coupled with machine learning classification algorithms have enabled quantification of gross motor performance at an accuracy that compares with human observers.^11^ Before implementing such methodology in wider clinical or research use, however, there is a need to confirm its replicability, accuracy, as well as practical feasibility and utility. These should be evaluated in settings that closely compare with the expected out-of-hospital user scenario: The wearable recordings are ideally performed by the parents without close supervision of research or clinical staff, and the analysis pipelines should provide results in a fully automatic manner from data collection to final scoring to avoid errors or biases by the human(s) in the loop.

Here, we aimed to study how well the early development of gross motor performance can be assessed out-of-hospital using wearable recordings^15^ that are analysed with a fully automated pipeline. This wearable solution is compared to an algorithmic analysis that detects both six postures (supine, prone or side lying, crawl, sitting, standing) and seven types of movement (still proto, elementary, fluent, pivot, roll, transition) for each second of infant recordings.^15^ These gross motor abilities link to many milestones^16^, ^17^, ^18^ though they represent only a low-resolution sub-selection of the vast array of possible motor abilities seen in infants; however, they are represented and reliably detected from such a multisensory wearable recording.^15^ The present study investigated three core questions: 1) How do the results replicate across infant cohorts. 2) How accurately can we estimate gross motor development as a function of infants’ age, and how does it compare with the accuracy seen in the universally used physical growth charts^19^ at the population level and 3) at the individual level. In order to allow comparison of the qualitatively different measures, motor performance and physical growth, we first transformed them into a common measure, developmental reference age prediction (DAP). Such transparent and direct comparison of functional vs. physical growth assessments could pave the way to using functional growth as a credible outcome measure in future clinical and scientific work.

## Methods

### Overview

**Fig. 1:**
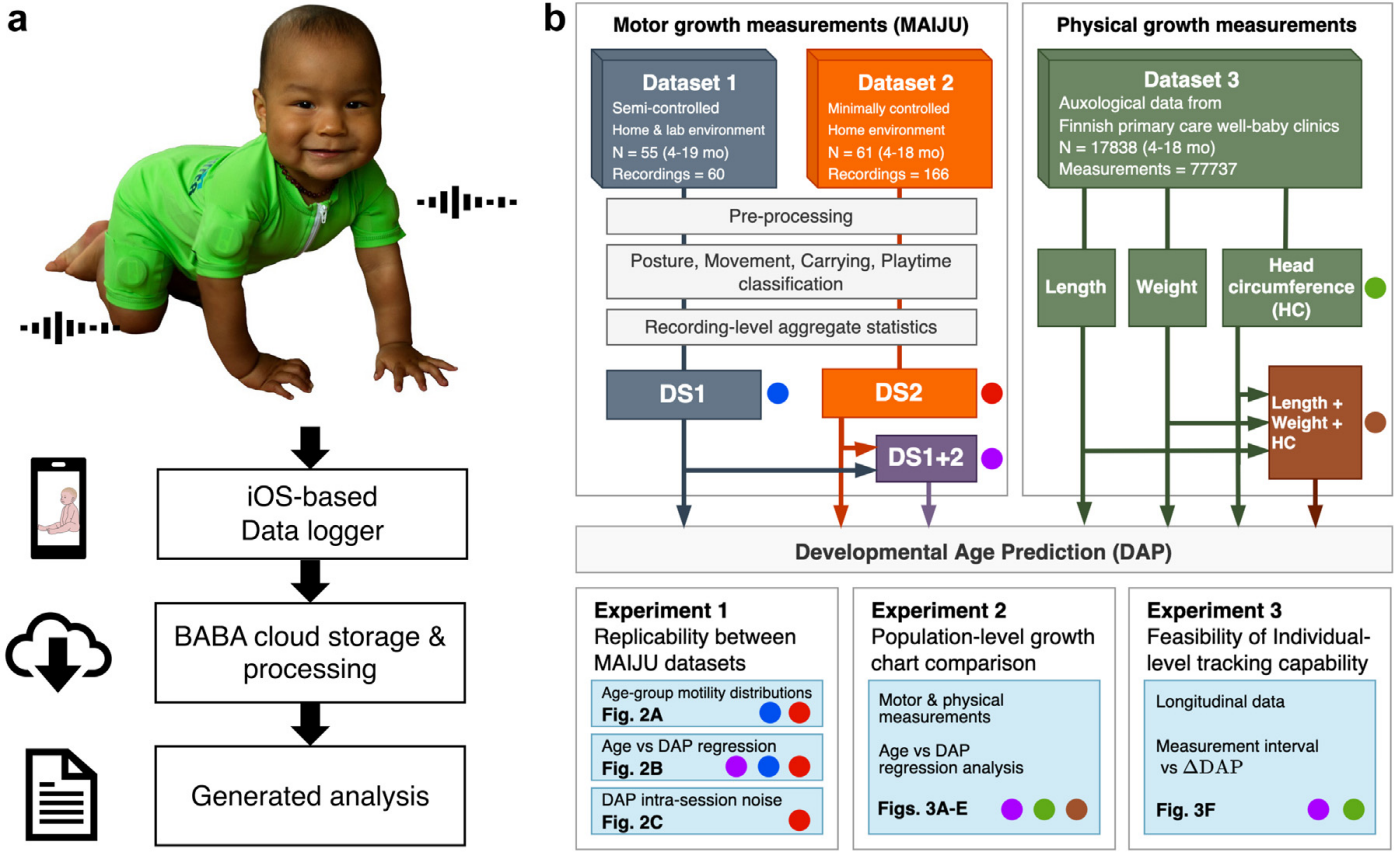
Study overview. a) A photograph of the MAIJU wearable in use by a 10-month-old infant and an overview of MAIJU's automatic data processing pipeline through a real-time data stream into mobile device, offline upload to computational server, and download of analysis results by the user. b) A block diagram of the study design. The coloured circles denote the respective datasets used in the experiments. Photograph is published with parental consent.

**Fig. 2:**
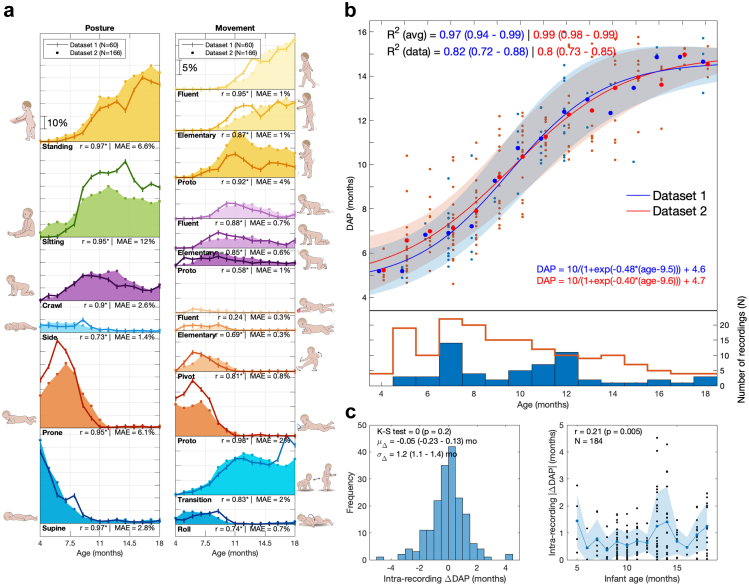
Assessing replicability between MAIJU cohorts. a) Comparison of age-group average distributions between DS1 and DS2 for posture (left) and movement (right). Pearson's r (∗ denotes statistical significance at p < 0.05) and the mean absolute error (MAE) are reported as performance metrics. b) Above: Comparison of DAP scores (small dots) as a function of age between DS1 (blue) and DS2 (red). A four-parameter logistic sigmoid regression (solid lines) and its ±1SD curves (coloured areas) are fit to the data. Goodness-of-fit of the regression model is estimated with R^2^ for the age-group averages (large dots) and all data points (small dots). Below: Count histograms for the number of recordings in each age group in DS1 (red line) and DS2 (solid blue). c) The measurement noise estimated as DAP score variability between consecutive full-hour epochs during a recording session. The histogram (left) shows all recording sessions together (N = 184), and the graph (right) presents the relationship of noise level with infants' Age.

The overall study design is presented in Fig. 1. We performed serial home recordings using a recently constructed multisensor wearable, MAIJU (Motor Assessment of Infants with a JUmpsuit).^15^ A novel dataset (DS2) was obtained using at-home recordings from a cohort of N = 61 typically developing infants (age 4.1–18.4 months), and the results were compared with a previously collected cohort of healthy infants (DS1; N = 55 infants, age 4.5–19.5 months).^15^ The MAIJU recordings were analysed using a fully automatic pipeline with deep learning -based classifiers^15^ to provide estimates of postures, movements, and carrying of the child at a timescale of seconds during time periods of the recording that were classified as “playtime” by another classifier based on parental reports. The statistical distributions of these measures can be aggregated to obtain a global measure of motor abilities, here expressed as developmental reference age prediction (DAP). The age-related changes in postures and movements were compared between cohorts (Fig. 2a), and the cross-validated DAP estimates were also compared after training on the different datasets alone or together (Fig. 2b; Appendix A1.3). Finally, the functional DAP-derived growth charts were compared with a large cohort (N = 17,838 infants) of dataset used for the Finnish national physical growth charts of length, weight and head circumference^20^; this aimed to directly compare measurement domains. In particular, we wanted to evaluate their relative accuracy in growth assessments, in order to facilitate future complementary use of both physical and functional measures.

### Recordings and analyses with the MAIJU wearable

#### Study cohorts (datasets, DS)

This study compares a novel prospectively collected cohort (DS2; N = 61 infants) to a previously collected dataset (DS1; N = 55 infants)^15^ which was used for training of the published posture and movement classifiers. Both datasets were recruited as a convenience sample from the volunteering population, and hence they cannot be considered as representative cohorts of the Finnish population. DS1 contains N = 55 typically developing infants (N = 60 recordings; total 79 h, age range 4.5–19.5 months). The novel cohort (DS2) was obtained from consecutively recruited infants into a study on the use of infant wearables for neurodevelopmental assessment (PILKE; clinicaltrials.gov #NCT05527080**)**. DS2 included a total of N = 166 sequential recordings from N = 61 typically developing infants (mean number of recordings 2.8 per infant; range 1–7) between 4.1 and 18.4 months of age. DS1 included multiple recordings from 5 infants (range 2–2, average 2), whereas DS2 included multiple recordings from 43 infants (range 2–7, average 3.3; Appendix A1.12). The recordings lasted from 1.4 to 16.9 h (average 7.8 h), with a total recording time in the cohort corresponding to 1279 h. Out of this time, 1025 h (0.5–16.8 h per infant, average 6.2 h) was deemed to have good enough signal quality (due to valid Bluetooth connection) for further analysis. A total of 328 h (0.1–6.4 h per infant, average 2 h) was depicted by the parents as “playtime”, which was used for the data analysis in the first phase; automated detection of free play time was used in the final results (see below). Playtime means periods where the infant is acting predominantly by him/herself, with minimal physical interference by the adults; however, adults or other children were allowed to be around and encourage the child in his/her typical spontaneous behaviour. Please see Appendix A1.1 for a detailed comparison of the datasets.

#### MAIJU recordings

We used an infant wearable, MAIJU (Motor ability Assessment of Infants with a Jumpsuit; Fig. 1a) that is essentially a commonplace full body overall garment equipped with four movement sensors, one in each sleeve.^11^^,^^15^ The sensors stream inertial measurement unit (IMU) data (3-axis accelerometer and gyroscope) at 52 Hz sampling frequency over a low-energy Bluetooth (BLE, v5.0) connection to a nearby mobile phone using a custom-built iOS application, “MAIJU logger” (Kaasa GmbH, Düsseldorf, Germany)*.* There was a difference in the recording settings between DS1 and DS2: Recordings in DS1 were performed by trained personnel either at infants' homes or at the lab in a playroom-like setting,^11^^,^^15^ hence considered a partly supervised setting. Only time periods of infants’ free play were recorded and analysed in DS1. In contrast, all infants in DS2 were recorded at home by the parents without a direct supervision by research personnel, hence considered a minimally supervised setting. The first recording of the infant was typically initiated during a visit to the lab (BABA center; affiliation a) to inform the parents about the study and to obtain a signed informed consent. The infants would then go home with the suit kept on until the evening. For the subsequent recordings, the sensor data collection in the MAIJU wearable was started in the lab and the wearable with the datalogger iPhone was sent to home in a bag (tablet sleeve) using an ordinary courier service. The parents dressed MAIJU on their infants and kept recording until evening sleep time. The parents were only given three instructions: i) to keep the recording mobile phone at a Bluetooth range, typically in the same room; ii) to allow and encourage the infant to play freely in the home environment without interruptions by the adults; iii) to write down approximate time windows of free play, which was later used for technical data verification and development of the pre-processing in our analytic pipelines.

Taken together, the study aimed to reach an ecologically relevant assessment of infants’ spontaneous motor activities. While the home is typically the ideal setting for each child, it is also apparent that substantial differences may exist between homes in terms of physical layout, family size, or child-relevant objects such as toys.

#### Automatic analysis pipeline of the wearable recordings

After the recordings, the data was uploaded from the recording device into a custom-built computational cloud using a web browser interface (link/resource available from the authors at request) which functions as a combined data storage and analysis platform. Detailed description of the deep learning -based classifier is published earlier.^15^ In brief, the data is first pre-processed, by segmenting into 2.3 s (120 sample) frames with 50% overlap (1.15 s; 60 samples), and segments of low signal quality are discarded from later analysis. These frames are then fed into three parallel operating neural network classifiers to make classifications for posture (7 categories), movement (9 categories), and carrying (i.e., free vs. caregiver assisted movement; binary). Free play time was finally classified with a binary support vector machine (SVM) classifier based on 10-min (520 frame) super segments (with 50% overlap) accumulated from the frame-level classifications outputs (see Appendix A1.4). The frames detected as low quality, infant carrying, or non-playtime were removed from further analyses. Finally, recording-wise distributions of postures and posture-conditioned movement categories were calculated and used as features in subsequent analyses.

Our preliminary analyses showed that the results are more reliable when the analysis pipeline is executed in a fully automated fashion without operator inputs (see Appendix A1.4 Fig. S4), such as manual addition of free play times taken from the parental notes. Therefore, the results hereafter are reported using analysis outputs from the fully automated pipeline, including an automated detection of infants’ free play.

#### Summarized measurement metrics with developmental age prediction (DAP)

It is well recognized that gross motor development is a multidimensional process where infants may acquire different abilities in varying order and varying times in a process that includes both individual and cultural factors.^21^ For the purpose of comparing between motor (functional) and physical growth charts, however, it was necessary to transform both motor and physical measures into a comparable quantity. Age is the key benchmark in all developmental assessments, hence it is intuitive to summarize measurements by using developmental age prediction (DAP; see Appendix A1.5). DAP was defined as *the statistical expectation of age that has most likely generated the given measurement*. In the present study, DAP was implemented with Gaussian Process Regression (GPR^22^). The DAP values can be then compared to the true chronological age at the time of the given measurement, and conversely, differences between DAP and chronological age reflect individual variation in infant development.

#### Statistics

Pearson's *r* was used to measure correlation in Experiment 1 (Fig. 2a and c), where statistically significant correlation is considered as p < 0.05. A four-parameter logistic sigmoid function (Appendix A1.6) with a least-squares fit was used to obtain average models between age and DAP. The goodness-of-fit of the sigmoid models was measured with the R^2^ and standard deviation (***σ***) (Appendix A1.7) relative to the raw data, age-group (by month) averages, and serial-measurement corrected version of the raw data. Linear mixed effects (LME) modeling was used for the serial-measurement correction (Appendix A1.8). The bootstrap method with N = 10,000 was used to obtain the 95% confidence intervals for the goodness-of-fit measures. The one-sample Kolmogorov–Smirnov (K–S) test was used to test for the null hypothesis that the data comes from a standard normal distribution (Fig. 2c).

### Experiment 1: assessing replicability between MAIJU cohorts

**Fig. 3:**
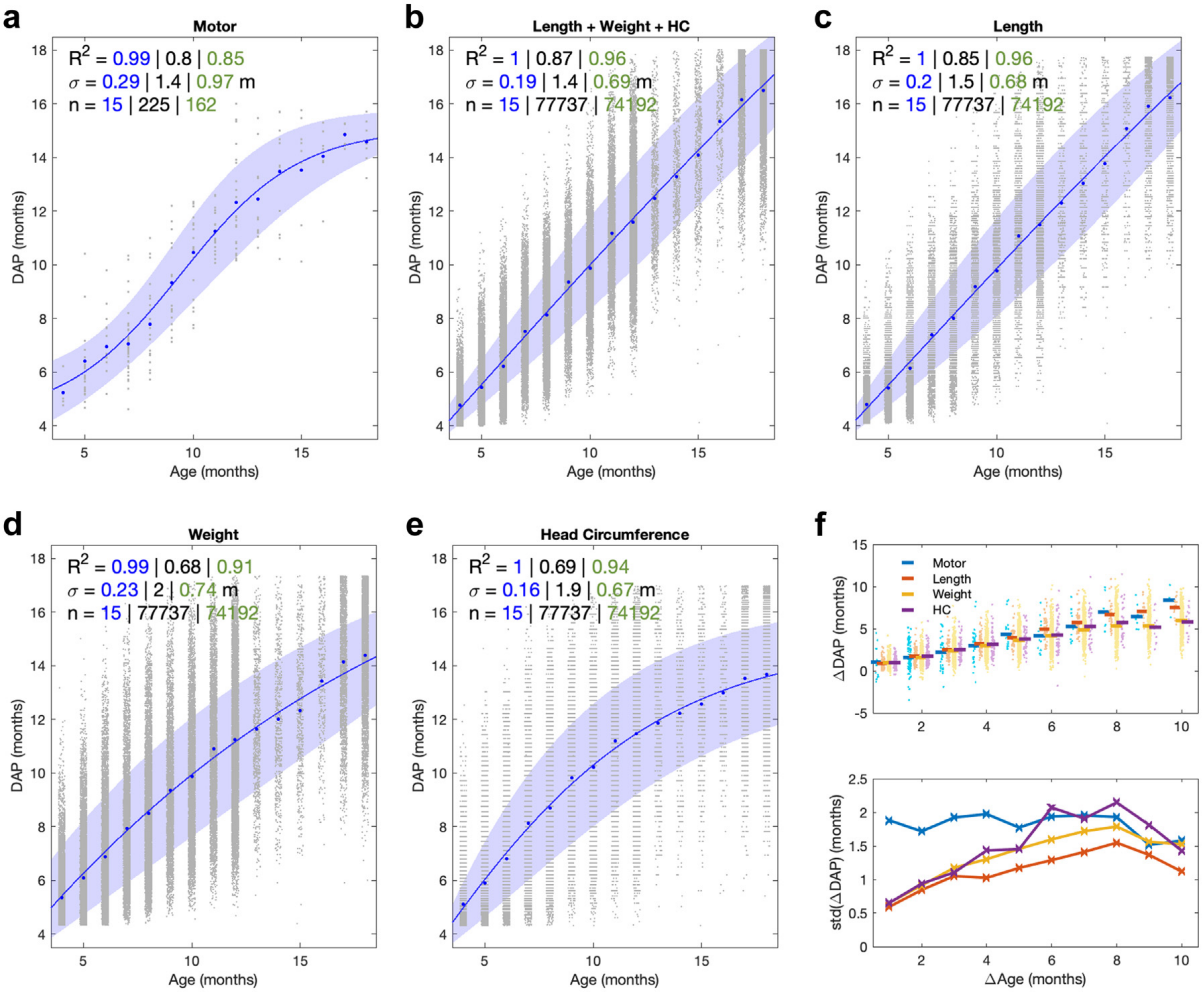
Growth charts of Developmental Age Prediction (DAP) scores for motor (a) and physical measures (b–e) between ages 4–18 months. The growth data are grouped into monthly bins and four-parameter logistic sigmoid regression models (solid lines; ±1SD depicted in colour) are fitted to growth data, followed by estimating goodness-of-fit in terms of R^2^ and standard deviation (***σ***) of the DAP scores. These measures are given in each plot for the age-group averages (blue), the raw data (black), and ID-controlled data (green). f) The effect of measurement interval on the developmental change measured by DAP. The upper graph shows how the observed advance in DAP scores generally correlates with the measurement interval with HC and is nearly identical for motor and length. The lower graph shows how, the variance (***σ***) in the DAP advance is higher with motor measures when using short measurement intervals; however, all modalities are comparable with longer measurement intervals.

To validate our previously published results with DS1,^15^ we compared the MAIJU findings from DS1 and DS2 as a function of age. First, the average age dependent distributions of different movement categories were compared visually (Fig. 2a), as well as by computing mean absolute error (MAE) and Pearson's correlation (r) between the findings from DS1 and DS2. Second, we compared the DAP measures (Fig. 2b) by computing the average regression model (four-parameter logistic sigmoid function; Appendix A1.6) between age and DAP for both datasets. The R^2^ measure was used to estimate the goodness-of-fit of the model against the raw data as well as age-group (by month) averages, including 95% confidence intervals using bootstrapping. The results in Fig. 3a are shown from Leave-One-Subject-Out (LOSO) cross-validated DAP scores trained with pooled DS1+2 data (see Appendix A1.3 for full results with cross-dataset DAP training).

Additionally, we estimated the inherent measurement noise level, or situational variability, in the DAP results from the DS2 recordings that contained >2 h of free playtime data (N = 75 recordings). These data were split into independent 1-h long segments to provide corresponding DAP estimates, from which the intra-session differences in DAP estimates were obtained (Fig. 2c; Appendix A1.9). The age dependency of the measurement noise level was examined by correlation with infant's age (Pearson; Fig. 2c). This test can be though to simulate test-retest variability. The effect of the recording length has been studied in a previous study,^15^ based on which the 1-h segment length was selected.

#### Physical growth data

Serial growth data of healthy infants were collected from the public primary care in the city of Espoo, Finland. The full database contained 561,392 length, weight and head circumference (HC) measurements from 75,810 subjects aged 0–24 months,^20^ and it can be considered representative of the paediatric population as it was collected to construct physical growth charts for the national health care use. Here, we used a subset of N = 77,737 serial measurements from N = 17,838 children aged 4–18 months that contained all three measures. Measurements were performed by primary nurses trained for auxological measurements during the regular, free-of-charge visits to public primary care child health clinics. Infants have regular visits at child health clinics at the ages of 1–2 weeks; 3–6 weeks; 6–8 weeks; at 2, 3, 4, 5, 6, 8, 10, 12, 18, and 24 months. Length, weight, and head circumference are measured using standardized techniques and calibrated equipment. Permission for the current study was obtained from Espoo Municipality Institutional Review Board. No contact was made with the study subjects since the data were handled anonymously.

#### Comparison of physical and motor growth data

For a direct comparability with the MAIJU-derived measurements, we constructed DAP scores also from all individual physical measures (length, weight, head circumference) as well as from the combination of all three measures. The direct physical measurements are presented in the Appendix A1.2 for comparison.

### Experiment 2: comparison of growth charts of motor and physical measures

We considered the chronological age of the typically developing children as the most accurate and unbiased benchmark for comparing growth charts from different measurement modalities.^19^^,^^20^ Therefore, we reasoned that the relative goodness of a measure in charting growth can be estimated by the fit of DAP scores to the age dependent regression model. It is important to note here that the nature of the underlying growth measure affects the shape of relationship between DAP and the chronological age, and that is not necessarily linear. For example, the motor scale in MAIJU saturates after the infant is fully able to walk fluently; such a motor growth chart is best modelled by a sigmoid function (see Figs. 2b and 3a), while length is an example of almost linear growth during the observation period. The goodness-of-fit is reported in terms of R^2^ with its standard deviation (***σ***) relative to the mean model. The analysis was first done using every measurement individually, but subsequent analysis with linear mixed effects modelling (see Appendix A1.8) considered serial recordings within an infant (N = 74,192 for physical, N = 162 for MAIJU), which accounts for the fact that infants typically develop in their own pace showing a consistent offset of the mean curve.

### Experiment 3: feasibility of individual-level longitudinal tracking

We investigated the behaviour of longitudinally obtained DAP scores for all measurement modalities as a function of the age difference between each two measurements. For all subjects with longitudinal recordings, each pair with measurement interval *Δage* = *t*_*1*_*−t*_*0*_*, Δ*DAP = DAP_t1_−DAP_t0_ was recorded (with *t*_*1*_ > *t*_*0*_), and we computed the means and standard deviations of *Δ*DAP in each monthly bin. The means represent the overall modelling capability of longitudinal measures in individuals, whereas the standard deviation reflects the level of noise in the overall trends.

#### Ethics

The study was approved by the Ethics Commission and fully executed by the Children's Hospital, Helsinki University Hospital, Helsinki, Finland. Written informed consent was obtained from the parents for all MAIJU recordings.

#### Role of funders

The funders did not have any role in study design, data collection, data analyses, interpretation or writing of the report.

## Results

### Experiment 1: assessing replicability between MAIJU cohorts

Distributions of postures and movement types showed expected,^15^ clear developmental changes (Fig. 2a). The characteristic evolution of posture types was: first a rapid decline of supine lying, followed by a comparable decline in prone lying, then a transient period with prominent occurrence of crawl posture, followed by a more persistent increase in sitting and standing, respectively. Conversely, evolution of the movement types within each posture context were clearly characterized by a transition from an initial “proto” movements towards more tentative elementary movements, until a fluent movement type is achieved.

Statistical comparison of age distributions of postures and movements showed a significant correlation (p < 0.05) between DS1 and DS2 in 16 out of the 17 measured motor categories (Fig. 2a; Posture categories: mean r = 0.9 range 0.74–0.97; Posture-conditioned movement categories: mean r = 0.78, range 0.24–0.98). The overall MAEs for posture categories were 5.2%-points (range 1.4–12.0) and posture-conditioned movement categories 1.1%-points (range 0.3–4.2). The largest discrepancies between DS1 and DS2 in terms of MAE were found within the posture category “sitting” and the movement category “standing-proto” (12%-p and 4%-p, respectively), though they still exhibited considerable correlation (r = 0.95 and 0.92, respectively). The only category with lower correlation between DS1 and DS2 was “prone-fluent” movement (r = 0.24, p = 0.4), which is likely due to its scarcity in the data (MAE = 0.3%-p). The comparison of age-grouped DAP measures (Fig. 2b) obtained from DS1 and DS2 show a striking resemblance with almost perfect overlap between the mean and ±1SD curves. The Pearson correlation between the age-group means was r = 0.98 (p = 10^−11^, N = 15) and all four regression model parameters overlapped within their 95% confidence intervals. The regression models explain 97% (R^2^; 94–99% CI 95) and 99% (98–99%) of the variance of age group means of DS1 and DS2, respectively. For DAP estimates from all the individual measurements, the corresponding values are 82% (R^2^; 74–85%) and 80% (72–88%). Taken together, these findings support the idea that MAIJU recordings provide measures of motor development that are highly replicable across datasets.

Finally, we assessed measurement noise by taking all available non-overlapping 1-h epochs (N = 39 infants, average 2.6 epochs (range 2–6) per recording) for which we computed intra-recording DAP variance (Fig. 2c; N = 184 combinations, average 2.5 combinations (range 1–15) per infant). The measurement noise was normally distributed (K–S test; p = 0.2), and the standard deviation of DAP measurement noise per recording is ***σ*** = 1.2/$\sqrt{2}$ that equals 0.85 months (see Appendix A1.9). The amount of measurement noise showed a small but significant age-dependent increase (Fig. 2c; Pearson's r = 0.21, p = 0.005), which likely reflects the increased variance of motor performance repertoire in the older infants.

### Experiment 2: comparison of growth charts of motor and physical measures

Inspection of the full cohort data (Fig. 3) shows that monthly mean values of all measurement modalities (motor, length, weight, HC, and physical combined) exhibit a very high goodness-of-fit (R^2^ = 0.99–1.00) between DAP and infant's age; however, there is also conspicuous variation in the shape of the overall growth trajectories between all measures.

Conversely, correlations of the individual growth measures with age were lower than population average measures: They were essentially comparable between motor, length and physical combined (Fig. 3a–c; R^2^ = 0.8, 0.85 and 0.87, respectively), and clearly lower (both R^2^ = 0.68) for weight and HC. Similarly, the variation of DAP against the average regression model was substantially lower for motor and combined physical measures (***σ*** = 1.4 months in both) as compared to weight and HC (***σ*** = 2.0 months in both). These results suggest that the measured modalities have the largest normal variability in weight and HC measurements. As the infants are well known to develop along their individual trajectories, implying a subject-specific offset relative to the population mean, we then estimated these age correlations by accounting for serial measures within an individual via introducing subject ID as a random variable using linear mixed effects modelling (see Appendix A1.8). This improved DAP goodness-of-fit against age in all modalities, with somewhat stronger effect in the physical measures (***σ*** range 0.68–0.72 months, R^2^ range 0.92–0.96) than the motor measure (***σ*** = 0.97 months, R^2^ = 0.85). Full details are shown in Fig. 3. Finally, we assessed the effect of sample size that differed by several orders of magnitude between motor and physical measures. Random sub-sampling of the physical measures for 500 iterations with sample size comparable to the available motor measures (N = 50 individuals; average 217 measurements (range 159–262)) showed that variation in the smaller sample sizes renders the age-correlations more comparable to those seen in the motor measures (see Appendix A1.10). Taken together, these results suggest that the statistics of gross motor performance can be represented as developmental trajectories despite the many sources of individual variance; thereby, the age-dependent tracking can be by and large comparable in accuracy with the established physical growth charts. However, as expected, there is somewhat higher measurement noise in the motor assessments, likely due to the situational variance in human behaviour.

### Experiment 3: feasibility of individual-level longitudinal tracking

Growth charts are typically used for longitudinal tracking of an individual's development over several time points, therefore we wanted to estimate how well each measure is able to reflect the respective growth between two consecutive measurements. Comparison of intervals from one to ten months shows that, on average, serial measures reflect development within individual infants in all cases (Fig. 3f, upper graph), seen as a monotonic relationship between average DAP scores and the time interval between measurements. However, motor measures have considerably higher variance compared to physical measures when the interval is less than four months (Fig. 3f, lower graph), while the variance becomes comparable between modalities with longer measurement intervals. In addition to the measurement noise (***σ*** = 0.85 months in motor DAP; see Experiment 1 above), this difference between motor and physical measures may be also related to the sigmoid-like shape in the MAIJU growth charts (Fig. 3a; Appendix A1.11): It indicates a sequence with slow-rapid-slow motor development, and consequently, measurements with shorter intervals may give more relative variance during the age ranges with a slower motor development.

## Discussion

We showed that objective and quantitative out-of-hospital assessment of infants’ developing motor abilities is possible by combining a multisensor wearable recording to a fully automated analysis pipeline. The findings from a novel cohort recorded without direct supervision validate previously published findings from a cohort recorded under partly controlled settings. Moreover, we show that the measures of gross motor abilities and DAP scores can be aggregated to motor growth charts at the population level, with an overall age modelling accuracy that is comparable to childhood physical growth charts.^19^^,^^20^ In the individual level, however, the assessment of growth trajectories of motor measures exhibits somewhat higher noise compared to the physical measures. The practical impact of this observation needs to be tested in different user scenarios. These findings together are perfectly aligned with the common knowledge that infant development follows a qualitatively predictable trajectory.^19^^,^^23^ We extend all prior literature by describing a widely scalable solution with automatic analysis pipelines that allows quantitative tracking of motor development at an individual level.

The analysis outputs from the automated pipeline provide a transparent, intuitive, and clinically explainable summary of gross motor behaviour. It also supports direct prediction of “motor age” in months, such as the DAP score in the present study, or it can be converted to a unitless score of motor performance, such as from 0 to 100 as in our previous study.^15^ A direct comparison of age-based modelling between motor and physical growth measures indicates largely comparable developmental trajectories in all measures despite their expectedly wide interindividual variance. Here, we found physical length to show the tightest link with age, which is compatible with the well-established use of stunted growth as a hallmark of compromised physical development.^24^ Age prediction from the other routinely used physical measures, weight, and HC, was closer to the modelling accuracy seen with measures of motor abilities, supporting its use as a complementary measure in developmental assessments. A particular challenge for any developmental tracking is to measure the rate of change over varying time intervals as many technical and biological factors may introduce “measurement noise” that obscure estimates of developmental progress. Our present findings suggest that such measurement noise may significantly interfere with assessing progress in motor abilities over brief time intervals up to few months; however, measurement noise becomes comparable between motor and physical assessments when longer intervals are used between assessments. Future studies are needed to standardize measurement practices to minimize any controllable noise, as well as to further evaluate its practical impact in different kinds of use cases.

Currently available clinical assessment of motor abilities is based on observing or surveying motor milestones^2^^,^^3^^,^^6^ or on performing standardized neurological assessment batteries, such as Hammersmith Infant Neurological Examination^7^ or Alberta Infant Motor Score.^3^ Milestones are practical in large scale population screening to obtain a rough and qualitative summary of a child's performance.^2^^,^^3^^,^^6^ However, motor milestones represent only a limited set of discrete performance categories with widely ranging reference values,^16^ which by nature compromises their utility in quantitative assessments and developmental tracking. The standardized assessment batteries, in turn, need trained professionals to collate empirical sets of motor items that can be clinically observed in a controlled environment, such as a doctor's appointment.^6^, ^7^, ^8^, ^9^ The results of these test batteries may show acceptable test-retest reliability,^6^ however they are resource-intensive, and always both partly subjective and semi-qualitative in nature, which inherently compromise their use in quantitative assessment and wider scale-up. The standardized assessments do also suffer from a lack of ecological validity because the study situations are typically unnatural from the infant's perspective. First reports have suggested that MAIJU-derived metrics correlate well with expert-driven neurological assessment,^11^^,^^15^ however future studies are needed to systematically validate and establish the links between the presently used motor metrics and many of the existing, established neurological assessment scales. It is also important to recognize that the MAIJU-derived metrics are qualitatively and phenomenologically different from the conventional assessment methods. Therefore, a direct comparison between MAIJU and the conventional methods may be useful for a general benchmarking purpose, whereas the actual utility of MAIJU is best determined by comparing MAIJU-derived results to the target clinical courses or situations.

Child's chronological age is the most important single benchmark in all paediatric assessments. All physical and functional development of a child is expected to follow predictable trajectories, and growth charts of many measures^19^^,^^25^ have been established to allow a standardized categorization in developmental assessment: delayed *vs.* typical *vs.* advanced. Recently, several machine learning -based measures of structural, functional, and molecular development have been proposed.^26^, ^27^, ^28^, ^29^, ^30^, ^31^ Our study shows that age prediction is feasible from both the physical and motor measures in a similar manner and at a comparable accuracy during at least the first two years of life. The accuracy of DAP from the motor growth chart was only marginally lower than the DAPs from the conventional physical growth measures. Prediction of child's age was clearly improved in all modalities when considering successive recordings of an individual, which is fully compatible with the clinical concept that individuals exhibit their own, trackable growth trajectories.

In the context of modelling individuals’ development, it is important to recognize the different shapes in childhood physical and motor development. While physical growth continues monotonically throughout childhood, motor growth is best viewed as a series of developmental phases with their characteristic motor categories and qualitative transformations between such periods. For instance, the motor description scheme underlying our current analysis pipeline^15^ will saturate around 18 months of age in typically developing infants; tracking further motor development will hence need training of new algorithms for motor abilities that are characteristic of an older age, such as running and jumping, or that altogether reflect different qualities of movement phenomena (e.g., macro activity type, fine-grained kinesiological qualities). The present implementation of motor growth charts should, therefore, be only considered for assessing motor behaviours that characterize typical progress during the first 18 months of life. Within these constraints, our work reports straightforward motor performance metrics for studying developmental cross–domain interactions, including physical growth, later neurocognitive outcomes, and/or environmental enrichment interventions.^32^ Recent studies have emphasized the important, likely causal role of early motor abilities in later emerging neurocognitive performance,^17^^,^^18^ suggesting that metrics of early motor abilities could be potentially used as a more comprehensive proxy measure of early neurocognitive development.

Our work has some limitations. The study describes a fully functional solution for at-home tracking of motor abilities, and it is already being used in some clinical trials; however, several technical, practical, and regulatory steps need attention before routine clinical use. First, wider user experience is needed from different environments and user groups to assess practical issues encountered during infant recordings or analysis pipelines. For instance, more prospective studies are needed to establish standardized recording guidelines (cf.^19^) to minimize confounding factors, such as the variability between homes and infants’ daily schedule, as well as to further evaluate their practical impact in different use cases. The test-retest variability within a recording frame of days, although simulated in the present study, still need proper investigation. Further studies are also needed to test the utility of MAIJU in early diagnostics and follow-up of neurodevelopmental compromise, or in measuring efficacy of early therapeutic interventions. A particular attention is needed to establish recommendations for detecting deviant development in successive recordings; for instance, prospective studies are needed to explore how MAIJU could be utilized in an early detection of cerebral palsy. Notably, the presently published cohorts cannot be regarded as normative data due to their limited size and uneven age distribution. Establishing reference charts of motor development will require concerted efforts and much larger data collection to ensure generalizability and replicability^33^ even if the overall findings may not necessarily change by inflating the cohort size *per se*.^34^ Such work is only possible via prospective, multicentre data collection, preferably from many cultural contexts.^6^^,^^8^^,^^35^ It is also essential to evaluate the need for culture-specific normative charts^2^^,^^36^ or possible updates over time.^37^ Additionally, an independent analysis is needed to assess the cost-benefit questions specifically in different health care settings; that is tightly linked to the broad need to build understanding about the added clinical or scientific value of this methodology relative to all the existing clinical methods and practices. Finally, prospective routine use requires registration of MAIJU as a medical device, which mandates an accurate definition of intended use cases,^38^ and building of trust among relevant communities in neurodevelopmental research and medical care.

Taken together, MAIJU solution overcomes many of the key bottlenecks in a scalable, objective, and quantified measuring of infants' motor development. First, it provides an ecologically valid assessment by bringing the recordings to a child's native environment, the home. Second, the recordings can be analysed with a fully automated analysis pipeline that removes user-related errors and harmonizes analytics across centres. Third, the results are transparent and intuitively meaningful, allowing for heuristic and evidence-based further use in many ways. Fourth, the analysis outputs were validated here with a prospective cohort acquired in a setting that closely corresponds to an intended out-of-hospital use case. Fifth, the motor measures were shown to be stable enough at individual level to allow for building motor growth charts akin to the physical growth charts that are now used as a proxy of paediatric health. Quantitative measures of early motor development may become a component in future clinical decision support systems,^39^ and a key outcome measure to benchmark any clinical trials during early childhood.

## Contributors

All authors read and approved the final version of the manuscript.

MA: All analyses, visualisation, writing–original draft, verified underlying data ET: Data collection, writing–review & editing AG: Data collection EI: Conceptualization AS: Data collection, data interpretation, writing–review & editing US: Data collection, data interpretation, writing–review & editing OR: Conceptualization, data interpretation, methodology, supervision, writing–review & editing LH: Conceptualization, methodology, supervision, data interpretation, resources, writing–review & editing SV: Conceptualization, methodology, supervision, data interpretation, resources, writing–original draft, verified underlying data.

## Data sharing statement

The data or materials for the experiments reported here can be made available at reasonable request and within relevant legal constraints.

## Declaration of interests

E.Ilén is the owner and CEO of Planno. Planno provides the product and process R&D services in textile-based products. The other authors have no conflict of interest to report.
