# Supplementary material for: Charting infants’ motor development at home using a wearable system: validation and comparison to physical growth charts
Source: eBioMedicine. 2023 May 1;92:104591. doi: 10.1016/j.ebiom.2023.104591 (PMC10176156; doi:10.1016/j.ebiom.2023.104591)
Supplement: Supplementary Appendix [file mmc1.docx]

**Supplementary Appendix A1**

This appendix is supplement to:

Airaksinen M, Taylor E, et al. Charting infants’ motor development at home using a wearable system: validation and comparison to physical growth charts.

Table of Contents

[A1.1. Comparison of the infant cohorts 2](#_Toc132275403)

[A1.2. Physical measurements, raw data 3](#_Toc132275404)

[A1.3. Comparison of GPR models used for analysing MAIJU recordings 3](#_Toc132275405)

[A1.4. Free play time classification 4](#_Toc132275406)

[A1.6. Sigmoid model fitting info 6](#_Toc132275407)

[A1.7. Performance metrics 6](#_Toc132275408)

[A1.8. LME compensation for serial recordings 6](#_Toc132275409)

[A1.9. Estimation of the measurement noise in MAIJU recordings 6](#_Toc132275410)

[A1.10. Effect of random sub-sampling of the physical measures 7](#_Toc132275411)

[A1.11. Visualization of serial recording trajectories in MAIJU recordings 8](#_Toc132275412)

[A1.12. Visualization of serial recordings by age at recording 9](#_Toc132275413)

# A1.1. Comparison of the infant cohorts

The table below summarizes clinical details of the datasets. Both datasets were collated from consecutive recordings with the MAIJU wearable in the same hospital (BABA center, Children’s hospital, Helsinki), but there were some differences, in particular in the recording setting: DS1 recordings were performed under direct supervision of the research personel, while DS2 recordings were performed by the parents at infant’s homes. In addition, there was a difference in the recruitment criteria. DS1 was recruited as a convenience sample of typically developing infants with various backgrounds, participating in a clinical study that focused on early brain compromise. DS2, in contrast, was recruited out-of-hospital by inviting typically developing infants to contribute to a study that develops infant wearables.

|  | DS1 (n=55) | DS2 (n=61) | Total (n=116) |
| --- | --- | --- | --- |
| Number of subjects | 55 | 61 | 116 |
| Number of recordings | 60 | 148 | 226 |
| Male/Female | 26 / 29 | 29 / 32 | 55 / 61 |
| % | 47 / 53 | 48 / 52 | 47 / 53 |
| Preterm/Full term | 9 / 46 | 6 / 55 | 15 / 101 |
| % | 16 / 84 | 10 / 90 | 13 / 87 |
| Age range (months) | 4.5 ­– 19.5 | 4.1 – 18.4 | 4.1 – 19.5 |
| Age mean (months) | 10.2 | 9.6 | 9.8 |
| Age std (months) | 3.6 | 3.6 | 3.6 |
| Multiple recordings (subjects) | 5 | 43 | 48 |
| Multiple recordings (recordings) | 10 | 148 | 158 |

# A1.2. Physical measurements, raw data

**
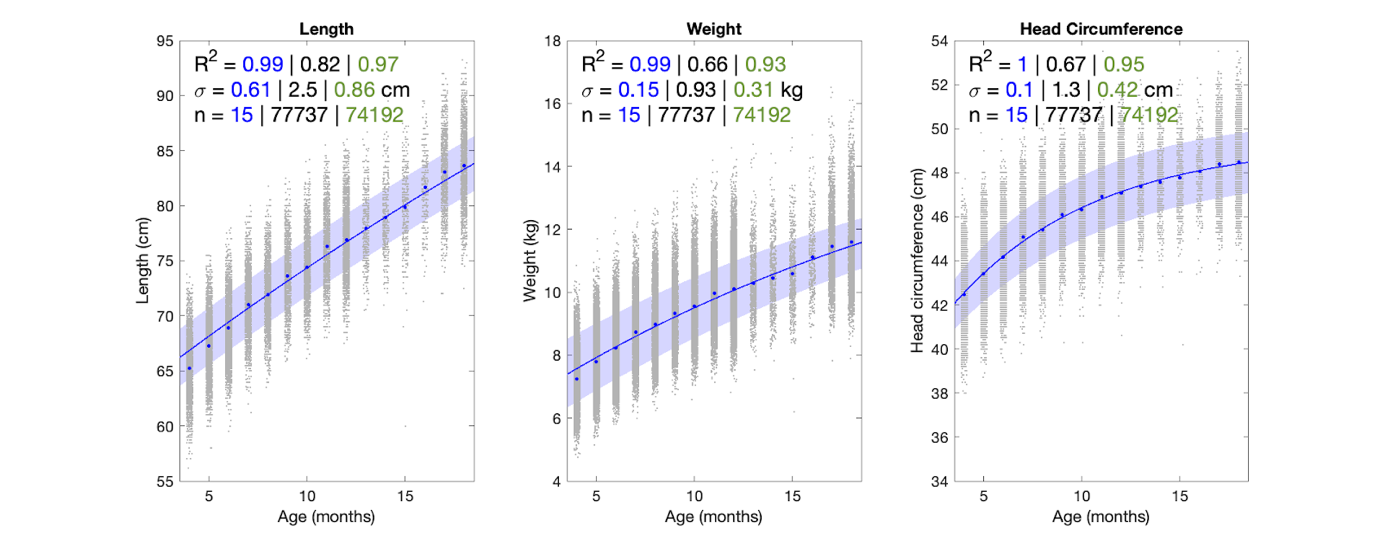
**

*Figure S1: Physical measurement growth charts obtained from the raw measurement values (i.e., without DAP; compare to Fig. 3C-E).*

# A1.3. Comparison of GPR models used for analysing MAIJU recordings

Our metric of motor performance (i.e., DAP) is based on a trained machine learning model, whose performance might depend on the combination of training and testing data. Typically, such models can be evaluated using a cross-validation approach, however an unseen, external data is needed to truly validate the algorithms’ performance. Here, we wanted to test all possible combinations of training and testing data for the GPR model for DAP (DS1, DS2, DS1+2). The histograms depict the age distributions available in each training cohort.

The graphs below show results after training the model with DS1 (left), DS2 (middle) or DS1+2 (right); the results are plotted using cross-validation approach for both DS1 and DS2 to fully demonstrate the replicability of the results. Taken together, the model trained with DS1+2 provides the best performance.

**
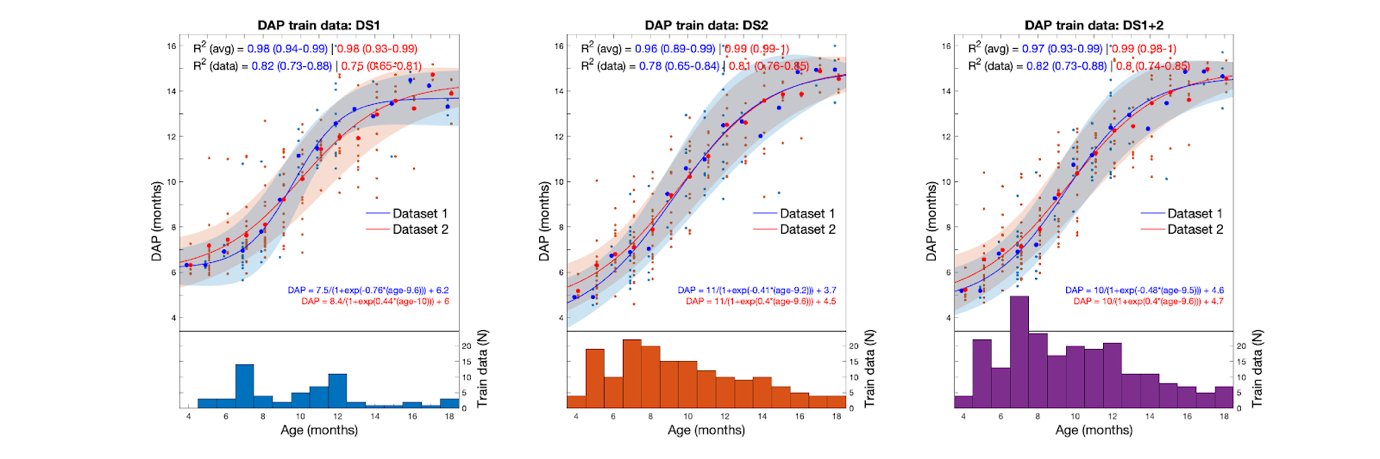
**

*Figure S2: Dataset comparison between DS1 and DS2 with varying DAP model train data (Compare to Fig 2B).*

# A1.4. Free play time classification

**
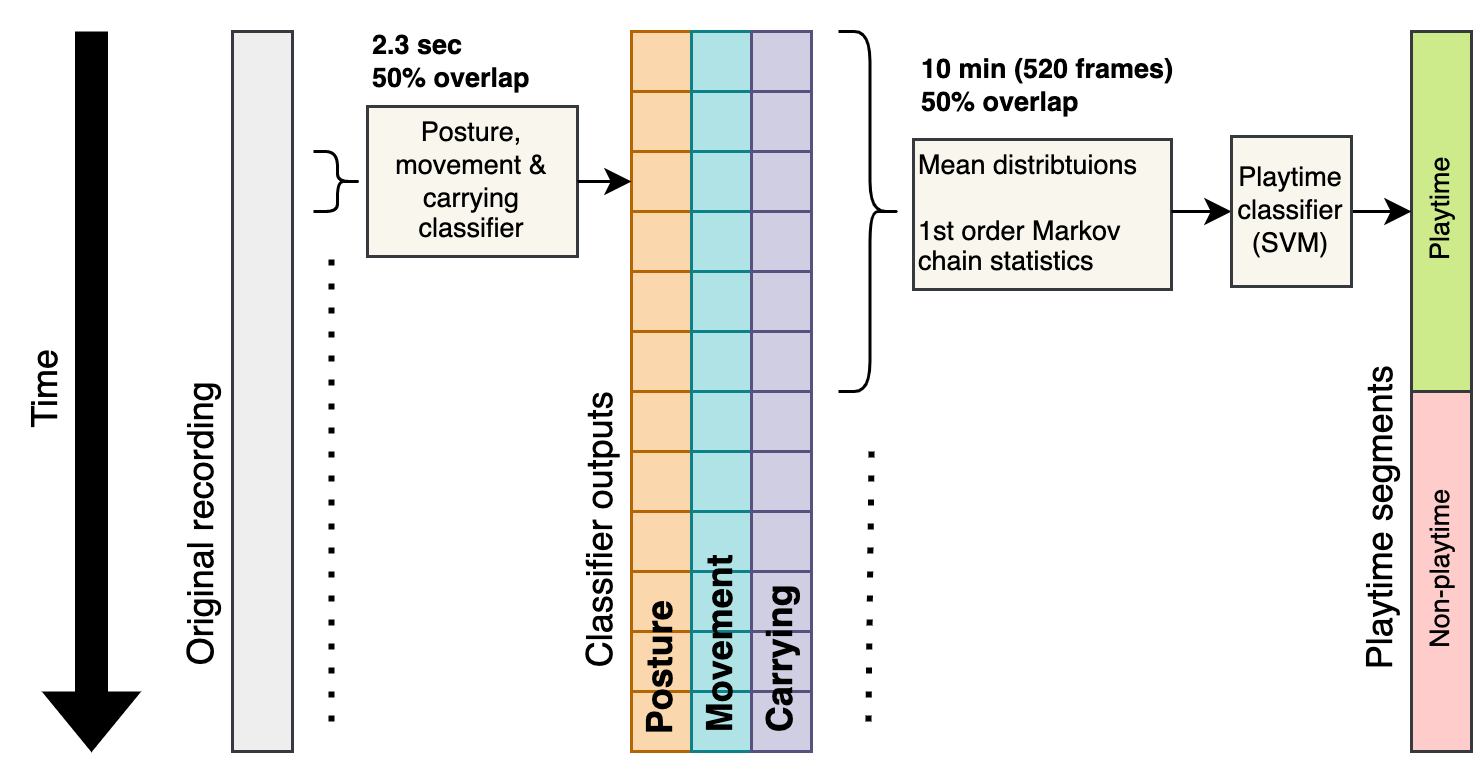
**

*Figure S3. Block diagram of the free play time classifier. The outputs from the posture, movement, and carrying classifiers are segmented into 10-minute macro-frames with 50% overlap. For each track, the mean distributions are computed, alongside the first order Markov chain transition statistics. All features are concatenated into a 52-dimensional feature vector representing the macro-frame. A support vector machine (SVM) classifier is trained to predict the binary decision whether a given segment is playtime or not. The classifier was trained with 15098 macro-frames of data from N=166 recordings from DS2 with the parent-reported playtimes as target outputs. The outputs utilized in subsequent analyses were obtained with 10-fold cross-validation at the recording level.*

**
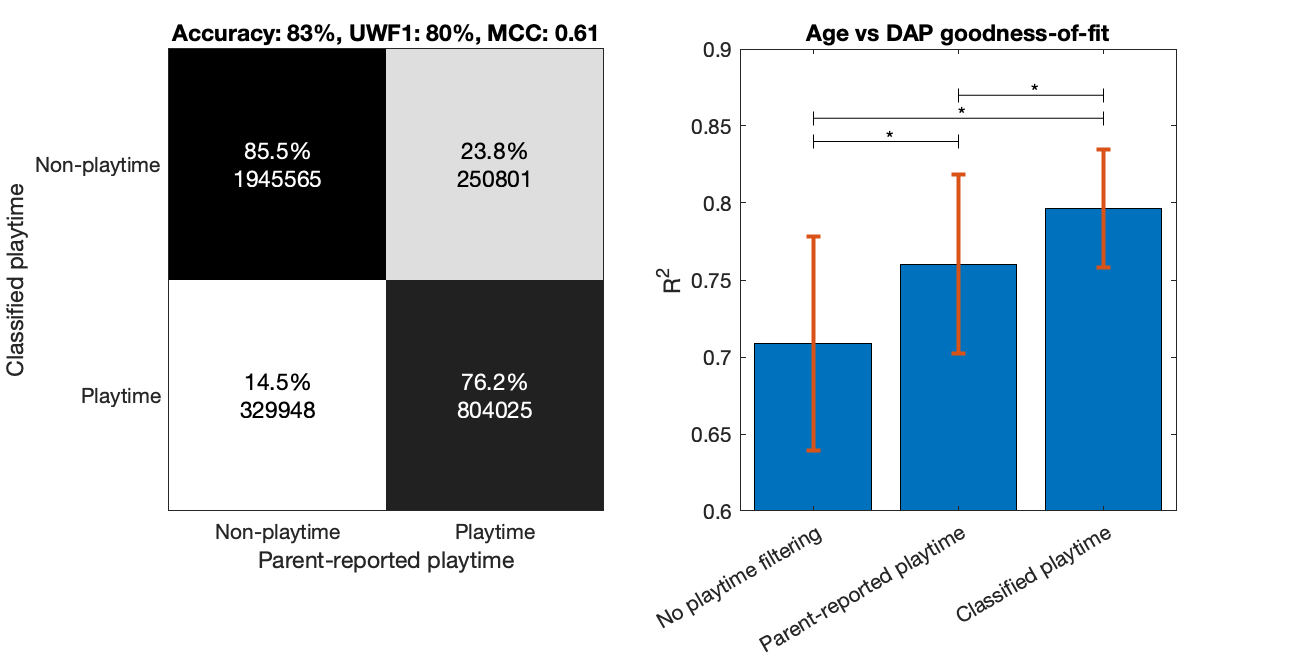
**

*Figure S4. Performance evaluation of the free play time classifier. Left: Confusion matrix between the parent-reported labels and the classifier outputs for single 2.3s frames (at 1.15 frames/s). The percentages inside the cells denote the recall values of the respective classifier decisions. The raw values denote the number of frames. The overall metrics reported are the accuracy, unweighted average F1-score (UWF1), and the Matthews Correlation Coefficient (MCC). Right: The effect of applying the free play time classifier into the computation of MAIJU distributions, in terms of the goodness-of-fit (R^2^) of the DAP scores versus the overall regression model. Results reported for three cases: no playtime filtering, parent-reported playtimes, and automatically classified playtimes. The red bars denote the 95% confidence interval for the R^2^ value obtained with the bootstrap method. The *’s denote statistical significance (p<0.05) of a two-tailed t-test between bootstrapped R^2^ differences testing for the null hypothesis that the respective difference is zero.*

**A1.5. Developmental age prediction (DAP)**

**
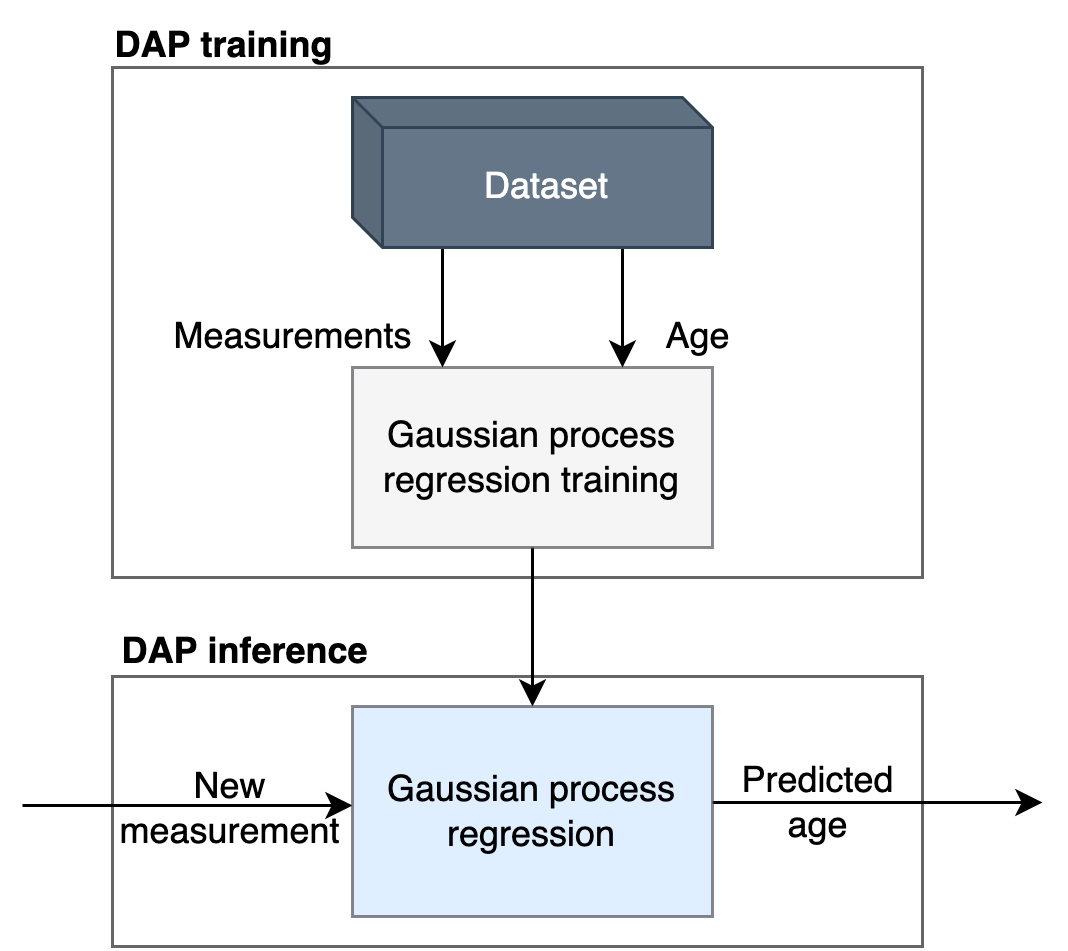
**

*Figure S5. Block diagram for the developmental age prediction (DAP) method. The measurements from a dataset are paired with the corresponding age at the time of the recording to train a Gaussian Process Regression (GPR) model. The model is used with unseen data to obtain the DAP score (aka the predicted age).*

The functional measurements m(x) at age x from MAIJU sensors and the aggregate M(x) from MAIJU posture and movement analysis pipeline can be considered as observable samples of a hidden (directly unobservable) generator function H(x) corresponding to the motor ability of an infant at age *x*. At the population level, H(x) is assumed to be normally distributed for any *x* with 𝜇(x) (monotonically increasing with x), and standard deviation 𝜎(x):

$$M(x) \sim H(x\mathbb{)=N(}\mu(x),\sigma(x))$$

An algorithm that searches for the most likely age *x’* (based on population-level statistics) responsible for generating the observation M(x) can be used to approximate an algorithm that searches for x’ based on H(x). Thus, the multivariate feature vectors M of MAIJU recordings are used to train a regression model that tries to predict the age based on the feature input (M_i_ → x_i_), from which the construction of a univariate growth chart as a function of age is possible (assuming that the phenomenology of H(x) allows it):

Gaussian process regression (GPR) as implemented by MATLAB Statistics & Machine Learning Toolbox was used for DAP. The model outputs for subsequent tests were obtained with cross-validation (LOSO for MAIJU, 10-fold for physical data). The GPR parameters were: 𝜎=2, matern52 kernel function, and constant basis function.

In the case of individual physical measures the “hidden” function H(x) (whose distribution DAP approximates) can be thought to be in relation to the measurement M(x) by a 1:1 mapping with an additive noise component caused by measurement noise 𝜎_meas_:

$$M(x) = H(x\mathbb{) + N(}0,\sigma_{meas})$$

# A1.6. Sigmoid model fitting info

The chosen least-squares regression model was a four-parameter (a,b,c,d) logistic sigmoid model:

$$y \approx f(x)_{a,b,c,d}= \frac{a}{1+e^{-b(x+c)}}+d$$

where *x* is infant age in months and y is the corresponding DAP score. The fit was implemented with MATLAB Curve Fitting Toolbox’s ‘fit’ function with nonlinear least squares fitting method, with starting point: a=12, b=0.7, c=-8.8, d=4. The logistic sigmoid was chosen as the model of choice because it can monotonically model exponential, linear, and exponentially saturating growth (and a sequential combination of them) at arbitrary scales.

# A1.7. Performance metrics

The fit of the regression models were compared to age-group (by month) averages, as well as to the original data with the R^2^ measure interpreted as the percentage of explained variance:

$$R^{2}=1-\sum_{i} ({y_{i}-f(x_{i}))}^{2}/ \sum_{i} ({y_{i}-E[y])}^{2}$$

where y is the DAP score, and f(x) is the corresponding regression model value at true age x.

The average standard deviation 𝜎 (aka rmse) of the DAP data around the sigmoid model was used as an additional measure for the goodness-of-fit. It was computed as:

$$\sigma=\sqrt{\frac{\sum_{i=0}^{N-1} (y_{i}-f(x_{i}))^{2}}{N}}$$

The mean absolute error between distributions p1 and p2 in Fig 2A was computed as

$$\mathrm{MAE} = \frac{\sum_{i=0}^{N-1} |p_{1,i}-p_{2,i}|}{N}$$

# A1.8. LME compensation for serial recordings

Linear mixed effects (LME; REF) modeling was used to account for a constant ID-specific random effect in the DAP scores for population-level tests. The LME model implementation was obtained from MATLAB Statistics & Machine Learning Toolbox with the formula:

$$f(x)_{a,b,c,d}\sim y+(1|ID)$$

using the parameters FitMethod: Maximum likelihood; Covariance pattern: FullCholesky; Optimizer: quasi-Newton.

# A1.9. Estimation of the measurement noise in MAIJU recordings

We reasoned that measurement noise equals to all the poorly identifiable or controllable variance in the analysis results; it arises from a multitude of mechanisms, which may relate to situational variance at the infant’s side (e.g.different play activities, vigilance state, immediate surroundings), recording technique (e.g. time-varying changes in the tightness of the MAIJU jumpsuit over infant’s body, or interruptions in the data streaming), or the analysis pipeline (e.g.accuracies of the serial classifiers in the pipeline). Their independent analysis may not be helpful for the practical purposes, thus we decided to estimate their combined effects in the typical recording scenarios. To this end, we compared consecutive full hour epochs of infants with multi-hour recordings, and we quantified such intra-session variance in motor measures. Assuming that the intra-session measurement noise is normally distributed (as shown by the Kolmogorov-Smirnov test in Fig 2C), the measurement noise can be approximated from the differences of independently drawn samples of a single session by dividing the observed standard deviation with $\sqrt{2}$.

Proof:

$$Var\left( X_{t0}-X_{t1} \right)=Var\left( X_{t0} \right)+Var\left( X_{t1} \right)-Cov\left( X_{t0},X_{t1} \right) = 2*Var(X)$$

$$\to\sigma(X) = \sqrt{Var(X)}= \sqrt{\frac{Var(X_{t0}-X_{t1})}{2}}= \frac{\sigma(X_{t0}-X_{t1})}{\sqrt{2}}$$

# A1.10. Effect of random sub-sampling of the physical measures

*Table S1: Mean and range for goodness-of-fit measures of physical growth DAP measures against average age-dependent trajectory. Results reported for 500 iterations of subsampling 50 individuals from the original physical measurement dataset (N=17,838). The mean and range for the number of recordings was 217 (159­–262).*

|  | Length | Weight | HC | Length+Weight+HC |
| --- | --- | --- | --- | --- |
| R^2^ | 0.85 (0.73–0.93) | 0.68 (0.50–0.82) | 0.69 (0.49–0.82) | 0.87 (0.78–0.94) |
| R^2^, LME | 0.96 (0.93–0.98) | 0.92 (0.86–0.96) | 0.94 (0.87–0.97) | 0.97 (0.93–0.98) |
| $\sigma$ | 1.48 (1.04–1.94) | 1.94 (1.59–2.22) | 1.88 (1.56–2.19) | 1.38 (0.95–1.81) |
| $\sigma, LME$ | 0.65 (0.41–0.93) | 0.71 (0.42–1.00) | 0.66 (0.39–0.93) | 0.64 (0.43–0.89) |

# A1.11. Visualization of serial recording trajectories in MAIJU recordings


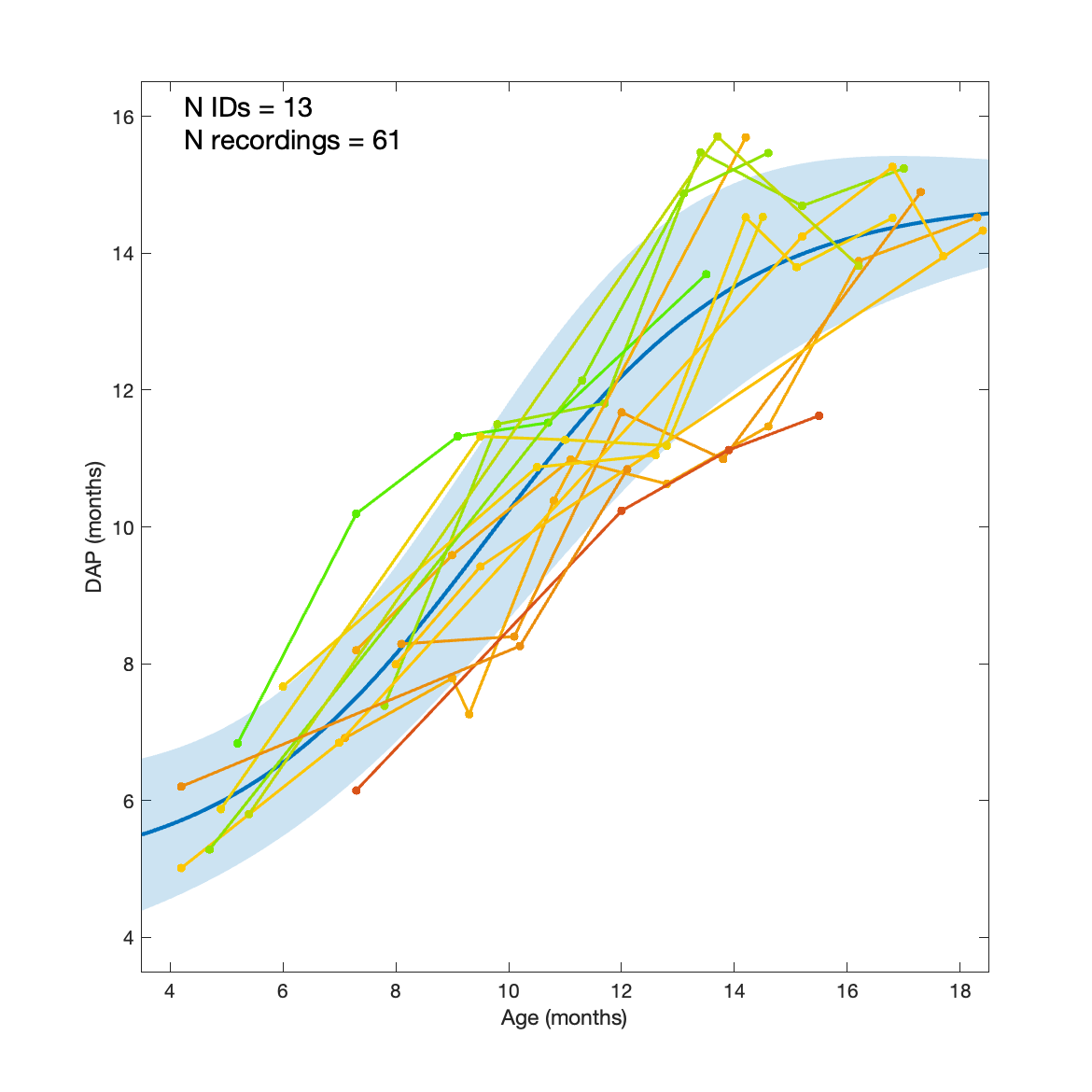


*Figure S6. Illustration of serial recording trajectories from DS2 from N=13 individuals. Inclusion criteria for the visualization was a minimum number of 3 recordings with a minimum difference of 6 months between the first and last recording age.*

# A1.12. Visualization of serial recordings by age at recording

**

*Figure S7. Illustration of the serial recordings as a function age (total N=48). DS1 (blue) has 10 serial recordings from 5 infants, whereas DS2 has 148 serial recordings from 43 infants.*
